# Personal earplug fit testing identifies below-target attenuation in nearly two-thirds of military aviation personnel: a retrospective analysis of results before and after individualized instruction

**DOI:** 10.3389/fpubh.2026.1955670

**Published:** 2026-09-30

**Authors:** Moshe Argaman, Oded Ben-Ari

**Affiliations:** 1 The Adelson School of Medicine, Ariel University, Ariel, Israel; 2 Carmel Medical Center, Haifa, Israel

**Keywords:** ear protective devices, hearing conservation, hearing protection fit-testing, military personnel, noise-induced hearing loss, occupational noise

## Abstract

**Background:**

Noise-induced hearing loss (NIHL) remains an important occupational health problem, especially in high-intensity noise work settings. Although disposable foam earplugs (DFE) are widely used, the protection they provide depends on how well they are fitted. We examined initial earplug attenuation in military aviation personnel, the change observed after individualized on-site instruction and repeat fitting among participants initially below target, and the association between previous instruction and baseline attenuation.

**Methods:**

This retrospective, single-center observational study analyzed routinely collected occupational-health fit-testing data from 183 military aviation personnel evaluated between 2020 and 2022. Mean age was 22.84 ± 6.23 years, and 91.3% were male. Earplug attenuation was measured using the 3M E-A-Rfit Dual-Ear Validation System. Participants with mean binaural attenuation below 20 dB received individualized on-site instruction and repeated the fitting procedure.

**Results:**

During initial fitting, 118 participants (64.5%) did not achieve the 20 dB target. In this group, mean binaural attenuation rose from 14.47 to 22.57 dB after instruction and repeat fitting, an improvement of 8.10 dB (*p* < 0.001). Participants who reported having received previous earplug-use instruction also had higher baseline attenuation than those without previous instruction (19.47 vs. 16.52 dB; *p* = 0.002).

**Conclusion:**

Quantitative fit testing identified inadequate initial attenuation in nearly two-thirds of the personnel assessed. Brief individualized instruction followed by repeat fitting was associated with a substantial improvement in attenuation among those initially below target. Incorporating personal fit testing and targeted instruction into occupational hearing-conservation programs may improve protection against NIHL.

## 1 Introduction

Noise is the most common occupational hazard (1, 2). Exposure to 85 dBA over an 8-h workday is generally considered the recommended occupational exposure limit, and each 3-dB increase in noise intensity halves the permissible exposure duration (1, 3, 4). Exposure to hazardous noise without effective protection may cause irreversible noise-induced hearing loss (NIHL), which is estimated to affect approximately 5% of the world’s population (2). NIHL can impair quality of life, communication, and daily and occupational functioning (5, 6).

Military aviation is a high-noise occupational setting in which ground and aircrew personnel may encounter aircraft noise levels of approximately 100 dB (7). In this setting, NIHL may impair communication and operational readiness, emphasizing the importance of effective hearing protection (5, 6). Prevention relies on reducing the duration and intensity of hazardous noise exposure and using effective hearing-protection measures when exposure cannot be adequately controlled (8). The 3M E-A-R Classic disposable foam earplug (DFE), commonly used in military aviation, carries a manufacturer-labeled Noise Reduction Rating (NRR) of 29 dB (9). However, the attenuation achieved by an individual user depends strongly on correct insertion and may be substantially lower when the earplug is fitted incorrectly (10, 11). Personal fit testing provides an objective, user-specific assessment of attenuation and can identify individuals who do not achieve a predefined protection target (11–13).

Previous studies have evaluated individualized or structured earplug instruction using approaches such as one-on-one or group teaching, written or video-based guidance, and device-assisted assessment of earplug fit, and have generally reported improved attenuation following instruction (10, 11, 14, 15). However, much of this evidence comes from controlled research settings or non-military populations, leaving limited information about real-world earplug performance during routine occupational use. In particular, it remains unclear how frequently users fail to achieve a predefined attenuation target when fitting earplugs as usual and what attenuation is observed after targeted instruction and immediate repeat fit testing. Data from military aviation populations are especially limited. This gap is particularly relevant in military aviation, where personnel may experience high-intensity noise exposure (7) while also needing to maintain communication and operational awareness. By analyzing objective personal fit-testing measurements obtained during routine practice, the present study provides real-world data on the prevalence of below-target attenuation, changes observed following individualized instruction and repeat fitting, and the association between previous instruction and baseline performance.

This study aimed to determine the proportion of noise-exposed military aviation personnel who did not achieve a predefined attenuation target during usual earplug fitting, to describe changes in attenuation following individualized instruction and repeat fitting among those initially below target, and to examine whether previous instruction was associated with baseline attenuation. Differences between two instruction protocols and according to hand dominance were examined as exploratory analyses.

## 2 Materials and methods

### 2.1 Study design and setting

This retrospective study analyzed routinely collected before-and-after earplug fit-testing data from noise-exposed Israeli Air Force personnel evaluated at the Israeli Air Force Aeromedical Center between 2020 and 2022 as part of hearing-conservation activities. Initial attenuation was measured during participants’ usual earplug fitting. Participants whose mean binaural attenuation was below the predefined 20 dB target received brief individualized on-site instruction and repeated the fitting procedure.

This testing-and-instruction sequence reflected the routine physiological-training workflow of the center and was not assigned by the investigators for research purposes; the present study retrospectively analyzed the data generated during this process.

Because personnel who did not achieve the attenuation target routinely received instruction and repeat testing, the dataset did not contain a comparable below-target group that underwent repeat testing without instruction.

### 2.2 Participants

This study included 183 military aviation personnel in noise-exposed ground and aircrew roles (91.3% male, 8.7% female; mean age 22.84 ± 6.23 years; range 18–48). Active-duty military aviation personnel who attended routine hearing-conservation activities during the study period and completed the earplug fit-testing procedure were eligible for inclusion. Participants were excluded if key fit-testing data were missing or incomplete, or if the procedure could not be completed sufficiently to allow attenuation analysis.

All personnel attending the relevant routine physiological-training sessions underwent earplug fit testing and were not selectively recruited for this study. The retrospective analysis included all available records with complete and usable fit-testing data; a separate log of incomplete or unusable records was not maintained.

### 2.3 Data collection procedure

Each participant completed a brief questionnaire (Appendix) and underwent an individual earplug fit test using the 3M E-A-Rfit Dual-Ear Validation System to obtain objective attenuation measures.

### 2.4 Subgroup classification for exploratory analyses

For exploratory analyses, participants were additionally classified according to instruction method and hand dominance. Instruction method was defined as either a full three-step instruction or a two-step instruction in which the final holding step was omitted. Instruction method varied by study period: participants evaluated in 2020–2021 received the two-step protocol, whereas those evaluated in 2022 received the three-step protocol. Hand dominance was obtained from the questionnaire and categorized as right- or left-handed. Hand-dominance data were available for 50 participants (44 right-handed and 6 left-handed), and these subgroup variables were used only for the relevant exploratory analyses.

### 2.5 Instruments

For this study, we used the 3M E-A-Rfit Dual-Ear Validation System, which assesses the effectiveness of earplugs by measuring their attenuation. The system consists of specialized software, a loudspeaker, and test earplugs equipped with two embedded microphone sensors: one sensor measures the external noise level, and the other measures the noise level within the ear canal. During testing, a test signal covering seven standard frequencies was presented for several seconds while the participant sat in front of the loudspeaker wearing the test earplugs. Attenuation (dB) was calculated as the difference between the recorded noise levels. The test earplugs used were the 3M E-A-R Classic DFE, corresponding to the model routinely issued to military aviation personnel. The E-A-Rfit system was factory-calibrated, and the 20 dB attenuation criterion used in the routine workflow was preconfigured in the system. The system then displayed a green pass indicator or red fail indicator relative to this threshold. This routine device output was used to determine whether additional instruction and repeat testing were required.

### 2.6 Procedure

Participants were tested individually in the same quiet room at the Aeromedical Center. All measurements were performed using the same E-A-Rfit testing setup and test-earplug model. Testing was conducted according to the manufacturer’s operating procedure for the E-A-Rfit system and the routine procedures used at the Aeromedical Center. Participants were seated facing the loudspeaker during testing. Two trained examiners were involved in data collection: one operated the E-A-Rfit system, and individualized instruction, when required, was provided by either examiner. Participants then completed a brief personal interview and inserted the test earplugs as they usually did during service, without additional fitting guidance. Participants were accustomed to using earplugs as part of their noise-exposed occupational activities; therefore, the initial and repeat fittings represented repeated performance of a familiar task rather than first-time exposure to earplug insertion. The E-A-Rfit system then measured the attenuation levels in each ear. Participants who did not reach a mean attenuation of at least 20 dB [NRR 29 dB declared by the manufacturer (9)] received individualized on-site instruction on proper earplug insertion and repeated the fit-testing procedure. The instruction included the following two or three steps:

1 Rolling and compressing the earplug between the fingers.
2 Stretching the ear canal by pulling the ear upward and outward with the opposite hand.
3 Inserting the earplug and holding it for a few seconds until it expanded.

Each session lasted 10 min. Repeat testing was performed immediately after instruction within the same session, after which the participants were debriefed.

### 2.7 Outcomes and variables

The primary descriptive outcome was initial target failure, defined as mean binaural attenuation below 20 dB during usual earplug fitting. Baseline binaural attenuation was calculated as the mean of the initial right- and left-ear attenuation values. Among participants initially below target, post-instruction binaural attenuation was calculated as the mean of the right- and left-ear attenuation values obtained immediately after instruction and repeat fitting. Change in attenuation was calculated as post-instruction minus baseline binaural attenuation. Post-instruction target attainment was defined as mean binaural attenuation of at least 20 dB. Previous earplug-use instruction was self-reported and categorized as yes or no.

### 2.8 Ethics approval and consent

The project was reviewed by the Academic Branch, Office of the Israel Defense Forces Chief Medical Officer, and was determined to be exempt from formal review by the IDF Medical Research Committee (Helsinki) on 13 June 2021 (exemption reference no. 6152). This exemption related to the retrospective analysis of routinely collected data. Earplug fit testing and individualized instruction had already been performed as part of routine hearing-conservation and physiological-training activities since 2020 and were not initiated for research purposes. The research component was limited to the retrospective collection and analysis of de-identified data generated during these activities.

### 2.9 Statistical analysis

The primary descriptive outcome was the proportion of participants who did not achieve the predefined 20 dB attenuation target during initial earplug fitting. Among participants initially below the target, the main pathway analysis compared mean attenuation before and after individualized instruction and repeat fitting for the right ear, left ear, and binaural mean using paired-samples t-tests. The main secondary analysis compared baseline attenuation between participants with and without self-reported previous earplug-use instruction using independent-samples t-tests. Comparisons between participants who received two-step versus three-step instruction, and analyses according to hand dominance, were considered exploratory. The instruction-protocol analysis compared post-intervention attenuation among participants who required intervention. Hand-dominance analyses compared ear-specific attenuation before instruction in the subgroup with available hand-dominance data and after instruction among those in this subgroup who required intervention. Missing values were not imputed.

All tests were two-sided, with statistical significance set at *p* < 0.05. Welch’s t-test was used when the assumption of equal variances was not met. Welch’s t-tests were used for all instruction-method comparisons to account for unequal group sizes and potential variance heterogeneity. Cohen’s d_z_ was calculated for the primary paired comparison, and Cohen’s d was calculated for the main secondary comparison and instruction-method comparisons. Mean differences for the primary paired comparison, main secondary comparison and instruction-method comparisons were estimated with 95% confidence intervals. No formal adjustment for multiple comparisons was applied; the comparison according to previous instruction was a secondary unadjusted analysis. Comparisons involving instruction method and hand dominance were exploratory, unadjusted, and intended solely for hypothesis generation; their *p* values were not interpreted as confirmatory. As this retrospective study analyzed routinely collected fit-testing data, no *a priori* minimum sample-size or power calculation was performed; the sample size was determined by the number of eligible personnel with complete and usable records available during the study period. Statistical analyses were performed using IBM SPSS Statistics, version 28 (IBM Corp., Armonk, NY, USA).

## 3 Results

### 3.1 Participant characteristics and initial target attainment

The study included 183 military aviation personnel serving in noise-exposed ground and aircrew roles. Participants were predominantly male (91.3%), with a mean age of 22.84 ± 6.23 years, and 67.0% served in ground crew roles. Overall, 118 participants (64.5%) did not achieve the predefined 20 dB attenuation target during initial fitting and underwent on-site instruction and repeat fitting.

### 3.2 Improvement in attenuation following individualized instruction and repeat fitting

Among the 118 participants initially below the target, mean attenuation across both ears increased following individualized instruction and repeat fitting, from 14.47 ± 5.08 dB to 22.57 ± 4.61 dB (mean difference, 8.10 dB; 95% CI, 7.22–8.98 dB, *p* < 0.001, Cohen’s d_z_ = 1.68). After instruction and repeat fitting, 85 of 118 participants (72.0%) achieved the 20 dB attenuation target. Significant improvements were observed in both the right and left ears, as detailed in Table 1. Figure 1 shows both the mean pre-to-post change and the distribution of individual changes in attenuation following instruction and repeat fitting.

**Table 1:** Change in attenuation following on-site instruction and repeat fitting among participants initially below the target (*n* = 118).

Measure	Pre-instruction attenuation, dB	Post-instruction attenuation, dB	Mean change, dB	Cohen’s dz	*p* value
Right ear	14.31 ± 6.96	22.72 ± 5.88	8.41 ± 7.34	1.15	< 0.001
Left ear	14.64 ± 6.01	22.42 ± 4.84	7.78 ± 5.84	1.33	< 0.001
Binaural mean attenuation	14.47 ± 5.08	22.57 ± 4.61	8.10 ± 4.81	1.68	< 0.001

Values are mean ± standard deviation (SD) attenuation levels in decibels (dB). Mean changes represent post-instruction minus pre-instruction attenuation and are reported as mean ± SD. p values were derived from paired-samples t-tests. Cohen’s dz represents the standardized mean paired change.

**Figure 1:**
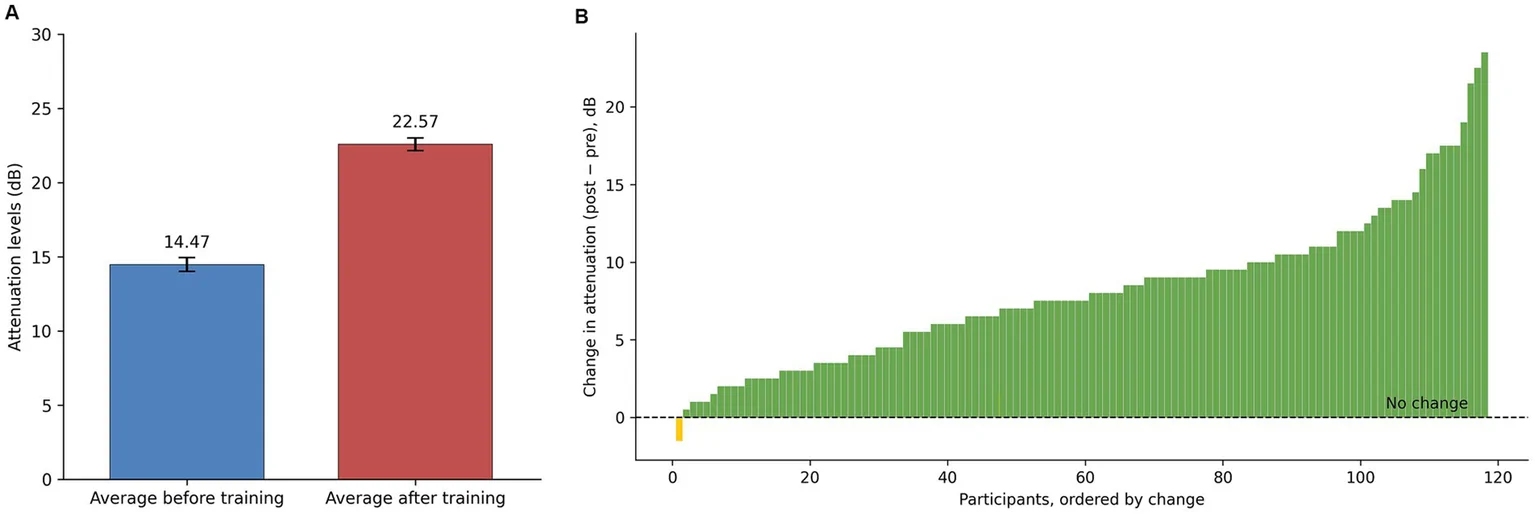
Mean and individual changes in attenuation after instruction. **(A)** Mean attenuation across both ears before and after instruction among participants who required intervention (*n* = 118). Error bars indicate the standard error of the mean (SE). **(B)** Individual changes in attenuation, with participants ordered by magnitude of change. Positive values indicate increased attenuation.

### 3.3 Association between previous instruction and baseline attenuation

Self-reported previous earplug-use instruction was associated with higher baseline attenuation. As detailed in Table 2, participants who reported previous instruction had a higher mean attenuation across both ears than those with no prior instruction (19.47 ± 6.30 dB vs. 16.52 ± 6.45 dB; mean difference, 2.95 dB; 95% CI 1.06–4.83 dB, *p* = 0.002, Cohen’s d = 0.46). The difference was larger and statistically significant for the left ear (19.94 vs. 16.29 dB; *p* < 0.001), but smaller and not statistically significant for the right ear (19.00 vs. 16.75 dB; *p* = 0.055). These comparisons are shown in Supplementary Figure S1.

**Table 2:** Association between previous instruction and baseline attenuation.

Measure	Previous instruction (*n* = 77), mean dB	No previous instruction (*n* = 106), mean dB	Mean Difference, dB (95% CI)	Cohen’s d	*p* value
Right ear attenuation	19.00 ± 7.60	16.75 ± 7.88	2.25 (−0.04 to 4.54)	0.29	0.055
Left ear attenuation	19.94 ± 6.17	16.29 ± 7.15	3.65 (1.65 to 5.65)	0.54	< 0.001
Binaural mean attenuation	19.47 ± 6.30	16.52 ± 6.45	2.95 (1.06 to 4.83)	0.46	0.002

Values are mean ± standard deviation (SD) baseline attenuation levels in decibels (dB). Mean differences and 95% confidence intervals represent previous instruction minus no previous instruction. *p* values were derived from independent-samples t-tests. Cohen’s d represents the standardized mean difference.

### 3.4 Exploratory analyses

The following subgroup analyses were exploratory, unadjusted, and intended solely for hypothesis generation.

#### 3.4.1 Comparison of two-step and three-step instruction

Among participants who required intervention, mean post-intervention attenuation was compared between those who received three-step instruction (*n* = 36) and those who received two-step instruction (*n* = 82). Because instruction method varied by study period rather than by random assignment, these comparisons should be interpreted cautiously. Mean binaural attenuation was higher in the three-step group than in the two-step group (23.90 vs. 21.99 dB; mean difference, 1.91 dB), but the difference was not statistically significant (*p* = 0.072; Cohen’s d = 0.42). Numerically higher attenuation was also observed in the three-step group for the right ear (24.08 vs. 22.12 dB; mean difference, 1.96 dB; *p* = 0.125) and the left ear (23.72 vs. 21.85 dB; mean difference, 1.87 dB; *p* = 0.089), although neither comparison reached statistical significance. These findings are presented in Table 3.

**Table 3:** Post-intervention attenuation by instruction method.

Measure	Three-step instruction (*n* = 36), dB	Two-step instruction (*n* = 82), dB	Mean change, dB (95% CI)	Cohen’s d	*p* value
Binaural mean attenuation	23.90 ± 5.68	21.99 ± 3.95	1.91 (−0.18 to 4.01)	0.42	0.072
Right ear	24.08 ± 6.65	22.12 ± 5.44	1.96 (−0.56 to 4.49)	0.34	0.125
Left ear	23.72 ± 5.81	21.85 ± 4.27	1.87 (−0.29 to 4.03)	0.39	0.089

Values are mean ± standard deviation (SD) post-intervention attenuation levels in decibels (dB). Mean differences and 95% confidence intervals represent three-step minus two-step instruction. *p* values were derived from Welch’s t-tests. Cohen’s d represents the standardized mean difference.

#### 3.4.2 Association between hand dominance and attenuation values

This analysis included 50 participants, comprising 44 right-handed (88%) and 6 left-handed (12%) individuals. Of these, 36 did not achieve the 20 dB threshold and received instruction- 31 right-handed and 5 left-handed. As shown in Supplementary Figure S2, initial attenuation did not differ significantly according to hand dominance in either ear or for binaural mean attenuation (all *p* > 0.35). However, after instruction, right-ear post-intervention attenuation was significantly higher in right-handed than in left-handed participants (25.19 vs. 17.20 dB; mean difference, 7.99 dB; *p* = 0.010). No statistically significant post-intervention differences were observed for the left ear (*p* = 0.483) or for binaural mean attenuation (*p* = 0.067). Given the very small number of left-handed participants, these findings should be considered hypothesis-generating and interpreted cautiously.

## 4 Discussion

### 4.1 Principal findings

The primary finding of this study was that 118 of 183 participants (64.5%), nearly two-thirds of the personnel (118/183, 64.5%) failed to reach the 20 dB target on their initial fit. After brief individualized instruction and a second attempt, mean attenuation in this group rose substantially by 8.1 dB. Mean attenuation increased from 14.47 dB to 22.57 dB, corresponding to a mean improvement of 8.10 dB. After instruction and repeat fitting, 72.0% achieved the 20 dB target. This change moved most trained personnel above the target and substantially narrowed the gap between real-world performance and the manufacturer-declared NRR of 29 dB. These findings are consistent with previous studies demonstrating that instruction can improve the effective fitting of disposable foam earplugs (10, 11, 14, 15).

In practical terms, an improvement of approximately 8 dB translates, under the 3 dB exchange rule, into more than a six-fold increase in allowable exposure time at a given noise level. We found that improper insertion can reduce attenuation by up to 50%, potentially resulting in inadequate protection against high noise levels.

These findings also indicate that usual, unassisted fitting of DFE was insufficient for many participants. Among those who required instruction, mean baseline attenuation was only 14–15 dB, well below both the 20 dB target and the manufacturer-declared performance. In a high-noise aviation environment, such suboptimal attenuation may leave personnel insufficiently protected despite wearing earplugs. In the current military aviation setting, there is no standardized formal protocol for earplug-insertion instruction, and most personnel receive only brief, informal guidance, often from firing-range instructors. This may contribute to suboptimal fitting. These findings emphasize that simply issuing earplugs is not enough: correct insertion must be taught and, wherever possible, verified.

### 4.2 Association between previous instruction and baseline attenuation

Participants who reported previous instruction achieved significantly higher baseline attenuation than those without such instruction (19.47 *vs*. 16.52 dB across both ears, mean difference, 2.95 dB, *p* = 0.002). Although this ~3 dB advantage may appear modest, it represents a meaningful reduction in effective noise exposure and corresponds to an approximate doubling of allowable exposure time. This association is consistent with the possibility that previously learned insertion techniques may persist over time (16), supporting the notion that “any instruction is better than none”. However, because previous instruction was self-reported and its timing, content, and quality were not documented, the present study cannot establish that instruction caused the observed baseline difference. These findings nevertheless support integrating regular, structured refresher sessions into instruction programs to help sustain effective insertion technique and long-term protection against NIHL.

### 4.3 Instruction method

We expected that a more detailed three-step instruction would yield greater attenuation than a simpler two-step method. Although post-instruction attenuation was greater in the three-step group (23.90 *vs*. 21.99 dB; mean difference 1.91 dB; 95% CI, −0.18 to 4.01 dB), this difference was not statistically significant (*p* = 0.072), and no significant ear-specific differences were observed. This result was contrary to our expectation, as the three-step method aligns with the manufacturer’s recommended fitting procedure; however, the more elaborate protocol was not shown to outperform the shorter one. Because instruction method varied by study period rather than being randomly assigned, this comparison is susceptible to temporal confounding, including possible differences in participant characteristics, examiner experience, or testing conditions, and cannot establish the relative effectiveness of the two protocols. Within these limitations, the observed numerical pattern may suggest that much of the improvement was already observed with the first two steps - rolling and compressing the DFE and adequately straightening the ear canal - whereas explicitly instructing participants to hold the earplug while it expands may provide a smaller or more variable additional benefit. In addition, the three-step subgroup was relatively small (*n* = 36) in comparison to the two-step subgroup (*n* = 82), limiting the precision of the comparison and precluding firm conclusions regarding the additional holding step.

### 4.4 Hand dominance and fine motor control

Since earplug insertion relies on fine motor coordination, attenuation in each ear could be expected to correlate with hand dominance. After instruction, right-handed individuals showed significantly higher right-ear attenuation than left-handed participants (25.19 vs. 17.20 dB), but no statistically significant differences were observed in other comparisons. This isolated finding does not establish a consistent association between handedness and earplug attenuation. The present findings do not provide sufficient evidence to support tailoring instruction according to handedness. Interpretation of the hand-dominance findings is limited by the very small number of left-handed participants, particularly the five left-handed participants with post-intervention measurements; therefore, this analysis should be considered exploratory and hypothesis-generating and should not be used to guide practice.

### 4.5 Comparison with previous research

Previous research has shown that instruction improves earplug attenuation, but most studies were limited by small samples, non-military populations, or controlled research settings rather than routine occupational-health practice (10, 11, 14, 15). For example, Murphy et al. reported that one-on-one experimenter instruction improved attenuation by approximately 5–8 dB compared with written or video instruction and noted that this magnitude was consistent with findings reported by Joseph et al. Joseph et al. demonstrated a significant instruction effect, with larger improvements after both individual and small-group instruction. Tsukada and Sakakibara similarly reported improved attenuation after individual education using an earplug-checking device. In our study, attenuation among participants who initially failed to achieve adequate protection increased from 14.47 dB to 22.57 dB after individualized on-site instruction, corresponding to a mean improvement of 8.10 dB. Our findings therefore align with the general pattern reported in previous studies while extending this literature by using an objective fit-testing system in a relatively large cohort of 183 active-duty military personnel and by simultaneously examining prior instruction, instruction structure, and handedness. By showing that individualized instruction followed by repeat fitting was associated with substantially improved attenuation, our findings further support the inclusion of practical insertion instruction and fit verification in military and occupational hearing-conservation programs.

### 4.6 Practical implications

Implementing a structured earplug-insertion instruction protocol together with personal fit testing in military aviation, integrated into basic training and reinforced periodically during service, may provide several health, economic, and organizational benefits: improved protection against NIHL and support for preserving hearing health and operational effectiveness; potential reductions in NIHL-related compensation claims and productivity losses (17); and a visible demonstration of organizational commitment to personnel well-being, which may enhance morale and reassure new recruits about noise-exposure risks.

Personal fit testing may also enable a targeted approach by identifying workers who do not achieve the required attenuation and directing instruction and repeat fitting to those most likely to benefit, rather than assuming that provision of earplugs alone ensures adequate protection. Because this testing-and-instruction pathway is brief and uses commercially available equipment, it may be adaptable to other high-noise workplaces, although its implementation and effectiveness should be evaluated in each occupational setting.

### 4.7 Limitations

This study has several limitations. Its retrospective, single center, two-year design under routine clinical conditions limited control over potential confounding and consistency of data collection. Because a separate log of incomplete or unusable fit-testing records was not maintained, their number could not be reconstructed, limiting assessment of potential selection bias. Previous instruction was self-reported, introducing recall bias regarding its timing, content, and quality.

Because only participants with baseline attenuation <20 dB received on-site instruction, the observed effect applies primarily to initially underprotected personnel and cannot be generalized to those already meeting the target. Selecting participants based on a low initial measurement also introduces the possibility of regression to the mean. Consequently, part of the observed pre-to-post increase may reflect statistical regression rather than the testing-and-instruction sequence itself, and the present design cannot quantify these contributions separately. The absence of a repeat-attempt control group without instruction limits causal interpretation. The testing sequence reflected routine practice rather than a research-assigned intervention, and participants were accustomed to using earplugs during their occupational activities; therefore, the repeat fitting represented another performance of a familiar task rather than a second-ever insertion attempt. Nevertheless, an effect of repeated fitting cannot be excluded, and the present design cannot determine how much of the observed change was attributable specifically to instruction versus repetition. The two instruction methods were implemented during different study periods rather than randomly assigned; therefore, their exploratory comparison may be affected by temporal confounding. The hand-dominance analysis was also limited by the small subgroup with available data.

No longitudinal follow-up was performed, precluding assessment of retention. Individual noise exposure and hearing outcomes were not assessed; the occupational relevance of the findings was inferred from the participants’ high-noise working conditions rather than from direct exposure measurements. Anatomical variation, manual dexterity, and the quality or recency of prior instruction may also have influenced performance. The predominantly young, male military aviation cohort may limit generalizability to other occupational populations. Some personnel may intentionally under-insert earplugs to preserve communication and situational awareness, potentially reducing measured attenuation and supporting the need for communication-compatible hearing protection.

### 4.8 Future research

Future prospective studies should include a repeat-testing control group to distinguish instruction from practice, evaluate instruction in personnel both below and above the baseline attenuation target, and assess retention over time. More detailed documentation of previous instruction would clarify its contribution to baseline performance. Research should also evaluate communication-compatible hearing protection, including high-fidelity earplugs or electronic systems, that permits necessary communication while maintaining adequate attenuation.

## 5 Conclusion

Proper use of DFE is neither intuitive nor guaranteed by simple provision of the device. Personal fit testing identified below-target attenuation in nearly two-thirds of this cohort of noise-exposed military aviation personnel. Among participants initially below the target, brief individualized instruction followed by repeat fitting was associated with a mean attenuation improvement of 8.10 dB, and 72.0% subsequently achieved the 20 dB target. Previous instruction was associated with higher baseline attenuation, although causality and long-term retention could not be established. These findings support incorporating personal fit testing and targeted insertion instruction into occupational hearing-conservation programs. Prospective controlled studies are needed to determine the durability of these improvements and their effects on real-world noise exposure and hearing outcomes.

## Data Availability

The de-identified data that support the findings of this study are available from the corresponding author upon reasonable request, subject to applicable ethical, privacy, and institutional restrictions.
