# Supplementary material for: Personal earplug fit testing identifies below-target attenuation in nearly two-thirds of military aviation personnel: a retrospective analysis of results before and after individualized instruction
Source: Front Public Health. 2026 Sep 30;14:1955670. doi: 10.3389/fpubh.2026.1955670 (PMC13661490; doi:10.3389/fpubh.2026.1955670)
Supplement: Supplementary file 1 [file Supplementary_file_1.DOCX]

**Supplementary Figure S1. Comparison of Mean Initial Attenuation Values Based on Prior Training**

Bars show mean baseline attenuation during initial/intuitive earplug insertion. “Both ears” represents the average of right- and left-ear attenuation. No previous training: n=106; had previous training: n=77. dB, decibels. Independent-samples t-tests: right ear, p=0.055; left ear, p<0.001; both ears, p=0.002.

Attenuation levels (dB)

|  | No previous training | Had previous training |
| --- | --- | --- |
| Right ear | 16.75 | 19 |
| Left ear | 16.29 | 19.94 |
| Both ears | 16.52 | 19.47 |

**Supplementary Figure S2. Mean Attenuation Values by Hand Dominance Before and After Instruction**

Attenuation levels (dB)

|  | Left-handed | Right-handed |
| --- | --- | --- |
| Right ear before instruction | 19.33 | 19.04 |
| Left ear before instruction | 16.33 | 19.59 |
| Right ear post-instruction | 17.2 | 25.19 |
| Left ear post-instruction | 22 | 24 |
| Average both before instruction | 17.83 | 19.32 |
| Average both post instruction | 19.6 | 24.59 |

Hand-dominance data were available for 50 participants (44 right-handed and 6 left-handed); among them, 36 required instruction and had post-intervention values available. Bar values represent mean attenuation in dB. Error bars indicate SE.

**Appendix**

Personal information form and questions answered by each participant during personal information collection as part of the study

1. Age:
2. Gender:
3. Role:
4. Dominant hand:
5. Have you received instruction on how to fit earplugs in the past?
6. If you answered positively to the previous question, when did the instruction take place? By whom?
7. Are you aware that noise exposure can cause hearing damage?
8. Rate your concern about hearing damage resulting from noise exposure on a scale of 1 to 5 (1 - not concerned at all, 5 - very concerned).
9. Do you have any additional comments or questions?
